# Effectiveness of simulation-based nursing education depending on fidelity: a meta-analysis

**DOI:** 10.1186/s12909-016-0672-7

**Published:** 2016-05-23

**Authors:** Junghee Kim, Jin-Hwa Park, Sujin Shin

**Affiliations:** The Catholic University of Korea 222, Banpodae-ro, Seocho-gu Seoul, 06591 Republic of Korea; Catholic University of Daegu, 33, Duryugongwon-ro 17gil, Nam-gu, Daegu, 42472 Republic of Korea; Ewha Womans University, 52, Ewhayeodae-gil, Seodaemun-gu, Seoul, 03760 Republic of Korea

**Keywords:** Nursing education, Patient simulation, Educational models, Meta-analysis

## Abstract

**Background:**

Simulation-based nursing education is an increasingly popular pedagogical approach. It provides students with opportunities to practice their clinical and decision-making skills through various real-life situational experiences. However, simulation approaches fall along a continuum ranging from low-fidelity to high-fidelity simulation. The purpose of this study was to determine the effect size of simulation-based educational interventions in nursing and compare effect sizes according to the fidelity level of the simulators through a meta-analysis.

**Method:**

This study explores the quantitative evidence published in the electronic databases EBSCO, Medline, ScienceDirect, ERIC, RISS, and the National Assembly Library of Korea database. Using a search strategy including the search terms “nursing,” “simulation,” “human patient,” and “simulator,” we identified 2279 potentially relevant articles. Forty studies met the inclusion criteria and were retained in the analysis.

**Results:**

This meta-analysis showed that simulation-based nursing education was effective in various learning domains, with a pooled random-effects standardized mean difference of 0.70. Subgroup analysis revealed that effect sizes were larger for high-fidelity simulation (0.86), medium-fidelity simulation (1.03), and standardized patients (0.86) than they were for low-fidelity and hybrid simulations. In terms of cognitive outcomes, the effect size was the largest for high-fidelity simulation (0.50). Regarding affective outcome, high-fidelity simulation (0.80) and standardized patients (0.73) had the largest effect sizes.

**Conclusions:**

These results suggest that simulation-based nursing educational interventions have strong educational effects, with particularly large effects in the psychomotor domain. Since the effect is not proportional to fidelity level, it is important to use a variety of educational interventions to meet all of the educational goals.

## Background

Clinical education in nursing aims to integrate theoretical knowledge from books into practical knowledge in real-life situations and to help students develop their problem-solving skills. Due to rapid changes in clinical placements, patient safety issues, and ethical concerns, students’ direct experience with patient care and opportunities to handle problem-based clinical situations have been diminished. Simulation-based clinical education is a useful pedagogical approach that provides nursing students with opportunities to practice their clinical and decision-making skills through varied real-life situational experiences, without compromising the patient’s well-being.

Simulation-based clinical education in nursing refers to a variety of activities using patient simulators, including devices, trained persons, lifelike virtual environments, and role-playing, not just handling mannequins [1]. With realistic clinical scenarios, simulation-based educational interventions in nursing can train novice as well as experienced nurses, helping them develop effective non-technical skills, practice rare emergency situations, and providing a variety of authentic life-threatening situations. The advantages of simulation-based educational interventions include the ability to provide immediate feedback, repetitive practice learning, the integration of simulation into the curriculum, the ability to adjust the difficulty level, opportunities to individualize learning, and the adaptability to diverse types of learning strategies [1].

Simulation can be described as a continuum ranging from low-fidelity simulation (LFS) to high-fidelity simulation (HFS) [2]. Various simulation methods can be adapted according to specific learning outcomes and educational levels. Dieckmann [3] warns against placing too much emphasis on having optimal equipment and surroundings that realistically replicate the clinical setting. The required learning outcomes must govern the choice of simulation method [4].

A number of research studies in nursing have evaluated the effectiveness of simulation-based educational interventions [5]. However, the reported effectiveness has varied according to the fidelity level of the simulators and the outcome variables. Issenberg et al. [1] found that HFS was effective for learning in medicine. However, their review was limited to HFS, medical education, and learner outcome variables, and did not compare simulation methods. Therefore, a meta-analysis synthesizing the results of these studies is needed to provide important insights into the level of simulation fidelity that is most effective for educational use.

The aims of this study were to determine the effect size of a simulation’s impact on nursing education and compare effect sizes according to the fidelity level of the simulators used.

## Method

This study was planned and conducted in adherence to PRISMA standards [6] of quality for reporting meta-analysis. We also considered the PRISMA criteria based on the PRISMA 2009 checklist in reporting each section, such as introduction, methods, results, and discussion.

### Study selection

Studies published between January 1995 and July 2013 were identified by conducting an electronic search of the following databases: EBSCO, Medline, ScienceDirect, ERIC, RISS, and the National Assembly Library of Korea database. The literature search was limited to articles published in English or Korean and was conducted using combinations of the keyword phrases *nursing, simulation, human patient,* and *simulator.* A total of 2279 potential studies were identified. Titles and abstracts were reviewed for eligibility.

**Fig. 1:**
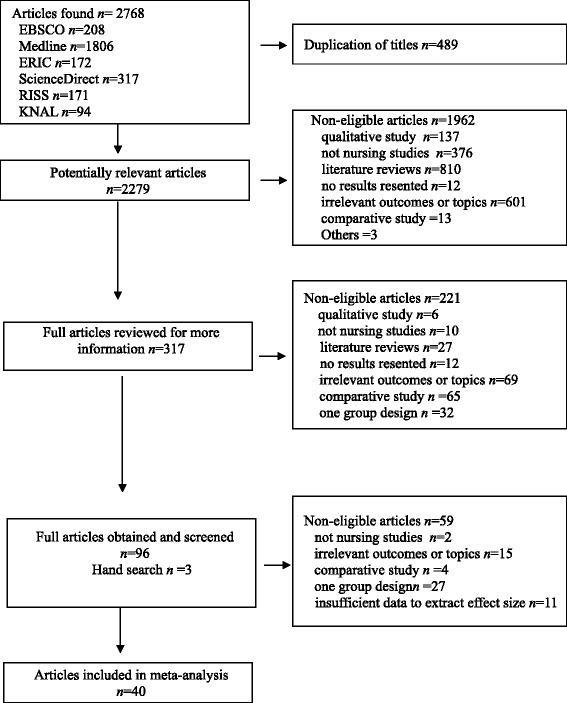
Flow of study analysis through different phases of the meta-analysis

Relevant studies were screened for inclusion based on the following criteria: 1) the study aimed to evaluate the effectiveness of simulation-based education for nursing students, and 2) an experimental or quasi-experimental design was used. We excluded articles that did not report a control group or that tested the effectiveness of computer-based virtual patients. For abstracts that did not provide sufficient information to determine eligibility, full-length articles were retrieved. Disagreement on the inclusion or exclusion of articles was resolved by consensus. Of the potentially relevant 2279 articles, screening of the title and abstracts resulted in 317 relevant studies. After a review of these articles, 96 studies were retained and three articles included additionally via hand search. These 99 full-text articles were reviewed systematically to confirm their eligibility (Fig. 1).

**Fig. 2:**
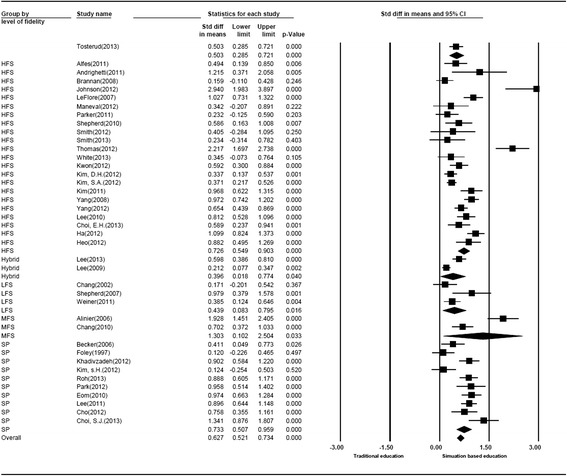
Forest plots for primary studies

### Criteria for considering studies for this review

In this study, assessment of the methodological quality of 40 selected studies was performed by using the Case Control Study Checklist developed by the Critical Appraisal Skills Programme (CASP) [7]. The CASP appraisal tool was designed to facilitate systematic thinking about educational studies. This tool contains 11 questions in three sections: (1) Are the results of the trial valid? (2) What are the results? (3) Will the results help locally? Most of the items were responded with “yes,” “no,” or “can’t tell.” The papers were assessed by two independent reviewers using the CASP checklist. Any disagreement that arose between the reviewers was resolved through discussion and consensus with a third reviewer. Forty studies met the inclusion criterion of nine or more out of 11 questions answered with “yes” and were consequently considered to be applicable to this review study. The inclusion criteria for this review were as follows:

#### Study participants

This study sampled pre-licensure nursing students, licensed nurses, or nurse practitioners.

#### Type of interventions

We defined *simulation-based educational intervention* as education involving one or more of the following modalities: partial-task trainers, standardized patients (SPs), full-body task trainers, and high-fidelity mannequins.

#### Types of outcome variables

Study outcomes included learning and reaction outcomes. Learning outcomes were categorized into three domains: cognitive, psychomotor, and affective.

### Data coding

The level of fidelity was determined by the environment, the tools and resources used, and other factors associated with the participants [8]. However, as to debriefing, a few selected studies do not indicate the method of debriefing they had used, making it difficult to categorize and discuss the effects of each debriefing method. Thus, we categorized fidelity level according to the physical equipment used. Fidelity level was coded as low, medium, or high according to the extent to which the simulation model resembled a human being, hybrid, or SP. LFSs were defined as static models or task trainers primarily made of rubber body parts [9, 10]. Medium-fidelity simulators (MFSs) were full-body manikins that have embedded software and can be controlled by an external, handheld device [10]. HFSs were life-sized computerized manikins with realistic anatomical structures and high response fidelity [11]. We also considered hybrid simulators, which combined two or more fidelity levels of simulation. As SP is a person trained as an individual in a scripted scenario for the purposes of instruction, practice, or evaluation [12], the use of SP was considered because of the different types of fidelity responses, such as body expressions and verbal feedback, which cannot be perceived in other simulation models.

The extracted data were coded by two researchers. A coding manual was developed in order to maintain the reliability of coding. The manual included information regarding effect size calculations and the characteristics of the study and the report. Differences between coders were resolved by discussion until a consensus was achieved.

### Data synthesis and analysis

The software Comprehensive Meta-Analysis version 2 (Biostat, Englewood, New Jersey) was used to conduct the data analysis. Effect size estimates were adjusted for sample size (Cohen’s d), and 95 % confidence intervals were calculated to assess the statistical significance of average effect sizes.

Fixed effects models assume that the primary studies have a common effect size. In contrast, random effects models attempt to estimate the distribution of the mean effect size, assuming that each primary study has a different population [13]. A test for heterogeneity of the intervention effects was performed using the Q statistic. As the results of the test for heterogeneity was statistically significant, we used the random effects models to accommodate this heterogeneity for the main effect and sub-group analyses. The planned subgroup analyses were conducted on evaluation outcomes.

## Results

### Study characteristics

We identified 2279 potentially relevant articles using the search strategy described above, of which 40 met the inclusion criteria. The characteristics of the 40 studies included in this meta-analysis are listed in Table 1. Twenty five of the 40 studies (62.5 %) used random assignment, whereas the remaining 15 (37.5 %) were nonrandomized. Half of the studies compared education using high-fidelity simulators with a control group. Ten studies (25 %) utilized standardized patients. Learners at various levels of training were represented.

**Table 1:** Characteristics of studies included in the analysis

Author (Year)	Country	Random assignment	Sample size experimental/control	Level of fidelity	Expertise-level of students
Tosterud (2013)	Norway	Y	29/28	HFS	1-3year
Alfes (2011)	USA	Y	29/34	HFS	1 year
Andrighetti (2011)	USA	Y	9/5	HFS	graduate
Johnson (2012)	USA	Y	19/16	HFS	graduate
LeFlore (2007)	USA	N	5/5	HFS	NP students
Maneval (2012)	USA	Y	13/13	HFS	graduate
Parker (2011)	USA	Y	18/23	HFS	2 year
Shepherd (2010)	UK	Y	9/15	HFS	3 year
Smith (2012)	USA	Y	16/17	HFS	3 year
Smith (2013)	USA	N	36/20	HFS	4 year
Thomas (2012)	USA	N	14/10	HFS	3-4year
White (2013)	USA	Y	16/38	HFS	4 year
Brannan (2008)	USA	N	54/53	HFS	
Kwon (2012)	Korea	Y	19/19	HFS	nurse
Kim, D. H. (2012)	Korea	N	69/62	HFS	4 year
Kim, S. A. (2012)	Korea	N	103/68	HFS	3 year
Kim (2011)	Korea	N	26/24	HFS	nurse
Yang (2008)	Korea	N	92/75	HFS	2 year
Yang (2012)	Korea	N	94/91	HFS	3 year
Lee (2010)	Korea	Y	35/34	HFS	1 year
Choi, E. H. (2013)	Korea	Y	32/33	HFS	2 year
Ha (2012)	Korea	Y	60/58	HFS	3 year
Heo (2012)	Korea	Y	26/31	HFS	3 year
Lee (2013)	Korea	Y	96/84	SP/LFS	2 year
Lee (2009)	Korea	N	141/142	SP/HFS	1 year
Chang (2002)	China	Y	14/14	LFS	nurses
Shepherd (2007)	Australia	Y	23/25	LFS	nurses
Weiner (2011)	USA	Y	23/23	LFS	nurses
Alinier (2006)	UK	Y	49/50	MFS	2 year
Chang (2010)	Korea	Y	20/20	MFS	nurse
Becker (2006)	USA	Y	47/82	SP	4 year
Foley (1997)	USA	N	28/38	SP	nurses
Khadivzadeh (2012)	Iran	Y	28/28	SP	midwifery students
Kim, S. H. (2012)	Korea	N	29/25	SP	3 year
Roh (2013)	Korea	N	35/39	SP	nurse
Park (2012)	Korea	Y	23/21	SP	4 year
Eom (2010)	Korea	N	31/31	SP	2&4 year
Lee (2011)	Korea	N	20/18	SP	2 year
Cho (2012)	Korea	Y	19/19	SP	nurse
Choi, S. J. (2013)	Korea	N	22/22	SP	3 year

### Overall analysis

When the studies were combined in the meta-analysis, high heterogeneity was observed (Q = 253.22, *P* < .001) (Table 2). The overall effect size for the random effects model was 0.70, with 95 % confidence intervals of 0.58–0.83 (Table 3) (Fig. 2). The possibility of a publication bias was minimal because the funnel plot appeared symmetrical.

**Table 2:** Results of the homogeneity test

N	*Q*	*p*-value	−95 % CI	ES	+95 % CI	SE
40	253.22	< .01	0.54	0.59	0.64	0.02

*N* number of studies, *Q* homogeneity statistic, *ES* effect size, *SE* standard error

**Table 3:** Overall result of the meta-analysis, using a random effects model

N	−95 % CI	ES	+95 % CI	SE
40	0.58	0.70	0.83	0.06

*N* number of studies, *ES* effect size, *SE* standard error

### Effect sizes by level of simulation fidelity

Studies using HFSs (0.86), MFSs (1.03), and SPs (0.86) had large effect sizes, whereas low-fidelity (0.35) and hybrid (0.34) simulation studies had smaller effect sizes.

### Reaction outcome according to fidelity level

The results of the sub-group analysis for reaction outcome according to fidelity level are shown in Table 4. The effect size of HFS on reaction was larger than that of LFS (Table 4).

**Table 4:** Effect sizes by level of fidelity, to evaluation levels

Outcomes	Type of fidelity	*k*	−95 % CI	ES	+95 % CI	SE
	HFS	77	0.67	0.86	1.05	0.09
	MFS	5	0.18	1.03	1.88	0.43
	LFS	13	0.18	0.35	0.52	0.86
	Hybrid	5	0.16	0.34	0.52	0.09
	SP	29	0.61	0.86	1.11	0.12
Reaction	HFS	5	0.41	0.64	0.87	0.11
	LFS	4	0.01	0.27	0.54	0.13
Cognitive	HFS	16	0.36	0.50	0.64	0.11
	MFS	1	−0.55	0.06	0.68	0.31
	LFS	1	−0.11	0.47	1.05	0.29
	SP	7	0.12	0.32	0.52	0.10
Affective	HFS	21	0.54	0.80	1.07	0.13
	MFS	1	−0.61	0.01	0.62	0.31
	LFS	4	0.06	0.39	0.71	0.16
	Hybrid	2	−0.03	0.35	0.75	0.20
	SP	9	0.51	0.73	0.95	0.11
Psychomotor	HFS	28	0.77	1.03	1.30	0.13
	MFS	3	1.41	1.76	2.11	0.17
	LFS	4	−0.05	0.38	0.82	0.22
	Hybrid	1	0.32	0.62	0.92	0.15
	SP	10	0.64	1.27	1.89	0.31

*k* number of effect size, *ES* effect size, *SE* standard error

### Learning outcome according to fidelity level

The results of the sub-group analysis for learning outcomes according to fidelity level are shown in Table 4. For cognitive outcome, which is a sub-domain of learning, the effect size was the highest for HFS (0.50), followed by LFS (0.47), SP (0.32), and MFS (0.06).

Regarding affective outcome, HFS (0.80) and SP (0.73) had the largest effect sizes, whereas LFS (0.39) and hybrid (0.35) simulation studies had smaller effect sizes. MFS (1.76), SP (1.27), and HFS (1.03) showed large effect sizes in the psychomotor domain (Table 4).

## Discussion

The present study provided meta-analytical data for evidence-based education through a comprehensive analysis of simulation-based nursing education with diverse backgrounds and characteristics. Compared with our previous article “Effectiveness of patient simulation in nursing education: meta-analysis” [14], the current study included an additional electronic search of Korean databases such as RISS and the National Assembly Library of Korea database. Through this process, 20 Korean papers were included additionally and half of papers were Korean. This could cause different result compared to previous one. In addition to including a reaction outcome according to fidelity levels, effect sizes based on outcomes and fidelity level were identified. A systematic search of the literature resulted in 40 published studies that were eligible for inclusion in this meta-analysis. These primary studies provided evidence of the effects of simulation-based nursing education in various evaluation and learning environments.

Random assignment studies accounted for 62.5 % of the studies included. This represents a noticeable increase in randomized research designs, which made up less than 30 % of studies in a systematic review conducted 10 years ago on HSF in medical education [1]. That review found that HFSs were used in 50 % of studies, and 25 % used SPs, which is similar to the findings of the study by Kim, Park, and Shin [15]. This confirms the relatively high usage of HFSs and SP in nursing education.

The medium-to-large effect size (0.70) suggests that simulation-based nursing education is effective. This is consistent with the findings of a study on health professional education [16], which reported that technology-enhanced simulation training produced moderate to large effects.

Regarding simulator fidelity level, HFS (0.86), MFS (1.03), and SP (0.86) displayed larger effect sizes compared to LFS or hybrid simulation. This result supports the findings of a previous meta-analysis of simulation in health professions, showing that HSF offers benefits over LFS [17]. However, these findings should be interpreted with caution. Recent studies suggest that the degree of realism required of a simulation is a function of the learning task and context, and can therefore vary widely for different areas of educational outcomes [17].

In the reaction domain, which includes satisfaction and learning attitudes, HFS had a larger effect size than LFS. Satisfaction levels are high among students participating in simulation learning that utilizes human simulators or SP [18]. Considering that problem-based learning (PBL) lessons were found to enhance student attitudes more than traditional lectures [19], student participation and actual activity appear to have positive effects on satisfaction and learning attitudes.

In the sub-group analysis for learning outcome according to fidelity level, the effect size was the largest for psychomotor outcome, followed by affective and cognitive outcomes. This result differs somewhat from the meta-analysis on the effects of PBL [19], in which effect sizes were the largest for psychomotor outcomes, followed by the cognitive and affective domains. This difference is interpreted as reflecting PBL’s emphasis on reasoning based on problems and cases, compared to the actual clinical practice emphasized in simulation-based learning.

Specifically, the effect size of cognitive outcome was the largest for HFS (0.50), while the order for affective outcome was HFS (0.80), followed by SP (0.73). In the psychomotor domain, the order was MFS (1.76), SP (1.27), and HFS (1.03). These results demonstrate that HFS and SP are effective in producing cognitive and affective outcomes; however, to achieve psychomotor learning outcomes, technical training using MFS would be more helpful, which concurs with the lack of positive association between fidelity and process skills [17].

However, the present study has the limitation of not considering learning-related factors in the analyses based on the fidelity level of simulators. Even though debriefing has become more crucial in simulation-based learning and the methods have diversified over the years, a few selected studies do not indicate the methods of debriefing they had used, making it difficult to categorize and discuss the effects of each debriefing method. This may be because it is customary to omit debriefing while learning from low fidelity simulations, especially for training simple nursing skills. As such, the present study has the limitation of not considering learning-related factors from debriefing at each fidelity level of simulators, including reflection, feedback, and a range of debriefing methods (self-debriefing, multimedia debriefing, and/or in-simulation instructor facilitated debriefing). In addition, we did not include studies published in languages other than English or Korean.

Despite such limitations, this study demonstrated that simulation-based nursing education has an educational effect, with particularly strong effects in the psychomotor domain. Since the effects are not proportional to fidelity level, educational interventions should be broad enough to satisfy educational goals, all of which are supported by the results presented above. In addition, a recent study reported that debriefing was the most important factor in simulation, with positive effects from self-debriefing and video-facilitated instructor debriefing [20]. Based on these findings, the clinical reflection process needs to be improved to increase the learning effects in the cognitive domain.

## Conclusions

Our results indicated that simulation-based nursing educational interventions were effective with particularly large effects in the psychomotor domain. In addition, the effect of simulation-based nursing education was not proportional to fidelity level. Therefore, it is important to use an appropriate level of simulation to meet all of the educational goals and outcomes.

## Abbreviations

HFS: high-fidelity simulation
LFS: low-fidelity simulation
MFS: medium-fidelity simulation
PBL: problem-based learning
SP: standardized patients

## References

[1] Issenberg SB, McGaghie WC, Petrusa ER, Lee Gordon D, Scalese RJ (2005). Features and uses of high-fidelity medical simulations that lead to effective learning: a BEME systematic review. Med Teach.

[2] Hovancsek MT, Jeffries PR (2007). Using simulations in nursing education. Simulation in nursing education: from conceptualization to evaluation.

[3] Dieckmann P, Dieckmann P (2009). Simulation settings for learning in acute medical care. Using simulations for education, training and research.

[4] Toserud R, Hedelin B, Hall-Lord ML (2013). Nursing students’ perception of high- and low-fidelity simulation used as learning methods. Nurse Educ Prac.

[5] Laschinger S, Medves J, Pulling C, McGraw R, Waytuck B, Harrison MB (2008). Effectiveness of simulation on health profession students’ knowledge, skills, confidence and satisfaction. Int J Evid Based Healthc.

[6] Moher D, Liberati A, Tetzlaff J, Altman DG (2009). Preferred reporting items for systematic reviews and meta-analyses: the PRISMA statement. Ann Intern Med.

[7] Critical Appraisal Skills Programme (CASP). Case control study checklist. From http://www.casp-uk.net/

[8] Dieckmann P, Gaba D, Rall M (2007). Deepening the theoretical foundations of patient simulation as social practice. Simul Healthc.

[9] Issenberg SB, Gordon MS, Gordon DL, Safford RE, Hart IR (2001). Simulation and new learning technologies. Med Teach.

[10] Seropian MA, Brown K, Gavilanes JS, Driggers B (2004). Simulation: not just a manikin. J Nurs Educ.

[11] Alinier G, Hunt B, Gordon R, Harwood C (2006). Effectiveness of intermediate-fidelity simulation training technology in undergraduate nursing education. J Adv Nurs.

[12] Robinson-Smith G, Bradley P, Meakim C (2009). Evaluating the use of standardized patients in undergraduate psychiatric nursing experiences. Clin Simul Nurs.

[13] Borenstein M, Hedges LV, Higgins JPT, Rothstein HR. Introduction to meta-analysis. West Sussex: Wiley; 2009.

[14] Shin S, Park J, Kim JH (2015). Effectiveness of patient simulation in nursing education: meta-analysis. Nur Educ Today.

[15] Kim JH, Park I, Shin S (2013). Systematic review of Korean studies on simulation within nursing education. J Kor Acad Soc Nurs Educ.

[16] Cook DA, Hatala R, Brydges R, Zendejas B, Szostek JH, Wang AT, et al. Technology-enhanced simulation for health professions education: a systematic review and meta-analysis. JAMA. 2011;306:978–88. http://dx.doi.org/10.1001/jama.2011.1234. 10.1001/jama.2011.1234 21900138

[17] Ilgen JS, Sherbino J, Cook DA (2013). Technology-enhanced simulation in emergency medicine: a systematic review and meta-analysis. Acad Emerg Med.

[18] Marken PA, Zimmerman C, Kennedy C, Schremmer R, Smith KV (2010). Human simulators and standardized patients to teach difficult conversations to interpersonal health care teams. Am J Pharm Educ.

[19] Shin I, Kim JH (2013). The effect of problem-based learning in nursing education: a meta-analysis. Adv Health Sci Educ Theory Pract.

[20] Levett-Jones T, Lapkin S (2014). A systematic review of the effectiveness of simulation debriefing in health professional education. Nurse Educ Today.

